# Accurate iodine quantification and residual error reduction with principal component analysis multimaterial decomposition using spectral CT

**DOI:** 10.1002/mp.70407

**Published:** 2026-04-10

**Authors:** Hamidreza Khodajou‐Chokami, Huanjun Ding, Sabee Molloi

**Affiliations:** ^1^ Department of Radiological Sciences University of California at Irvine Irvine California USA

**Keywords:** iodine quantification, residual error reduction, spectral CT

## Abstract

**Background:**

Multimaterial decomposition (MMD) in dual‐energy CT enables iodine quantification, critical for diagnostic applications. However, residual errors from noniodine materials in iodine maps limit accuracy, especially in complex thoracic regions and low‐dose settings.

**Purpose:**

To evaluate iodine quantification accuracy and residual error using a principal component analysis multimaterial decomposition (PCA‐MMD) algorithm on dual‐energy CT data across different phantom sizes and radiation dose levels.

**Methods:**

A thorax phantom containing iodine (2–20 mg/mL) and calcium (50–400 mg/mL) inserts was scanned on a clinical photon‐counting CT system. Three phantom sizes (small: 20.9 cm, medium: 27.3 cm, large: 33.2‐cm water‐equivalent diameter) were imaged at dose levels ranging from 3 to 55 mGy. The PCA‐MMD algorithm applies a principal component analysis (PCA) transformation followed by direct geometric estimation with barycentric coordinates, thereby avoiding matrix inversion instability. Iodine quantification was evaluated using linear regression, root mean square error (RMSE), and coefficient of variation (CV). Residual error in noniodine regions was expressed as a percentage of the minimum detectable iodine concentration. The algorithm's performance was also evaluated through a clinical proof‐of‐concept study involving five patients, comparing virtual noncontrast (VNC) images to true noncontrast (TNC) references.

**Results:**

PCA‐MMD achieved near‐unity regression slopes (0.98–0.99, $R^{2}\geq 0.996$) across all phantom sizes, reducing RMSE by up to 65% compared to the standard barycentric coordinate‐based MMD (0.10–0.39 vs. 0.20–0.72 mg/mL). Residual error was markedly lower with PCA‐MMD (0.7–1.6%) than with the standard barycentric coordinate‐based MMD (16.1%–54.9%) under identical conditions. At 3 mGy, PCA‐MMD achieved an RMSE of 0.60 mg/mL versus 0.95 mg/mL for the standard barycentric coordinate‐based MMD. In the clinical cohort, PCA‐MMD significantly improved VNC accuracy, achieving a mean RMSE of 15.5 HU compared to 20.9 HU for the vendor‐specific algorithm (p=0.012). Reproducibility was excellent, with 85% of the measurements showing a CV <2%.

**Conclusions:**

PCA‐MMD significantly improved iodine quantification accuracy and reduced residual error in both phantom and clinical settings. Its robustness across different dose levels supports its potential for clinical translation in quantitative dual‐energy CT applications.

## 1 INTRODUCTION

Dual‐energy computed tomography (DECT) provides spectral information by exploiting differences in x‐ray attenuation at two energy spectra, with photon‐counting CT (PCCT) extending this capability using energy‐resolving detectors,^1^, ^2^, ^3^, ^4^ enabling multimaterial decomposition (MMD) to generate quantitative maps for materials such as air, water, calcium, lipid, collagen, and iodine. Among these materials, iodine holds particular clinical significance due to its widespread use as a contrast agent. Accurate iodine quantification is critical for diagnostic applications including coronary CT angiography (CCTA), radiotherapy planning, tissue perfusion assessment, and tumor characterization.^5^, ^6^, ^7^, ^8^ However, the clinical utility of these iodine maps is often compromised by technical limitations in MMD algorithms, leading to significant diagnostic challenges.^9^, ^10^ Residual errors, particularly from dense materials like calcium and bone, can create false iodine signals that may mimic pathology.^11^ For instance, in CCTA, residual signals from calcified plaque can mask a critical stenosis or be misinterpreted as plaque enhancement, potentially leading to an incorrect assessment of coronary artery disease.^12^, ^13^ Similarly, in the thorax, a calcified lymph node may generate a false‐positive signal that can be misattributed to adjacent structures, potentially contributing to diagnostic confusion in vascular or perfusion assessments and risking inappropriate clinical management.^14^ Furthermore, inaccurate quantification, especially at the low concentrations relevant for perfusion imaging, can undermine the diagnostic value of the technique. In myocardial perfusion studies, these inaccuracies may lead to the misclassification of ischemic tissue, affecting treatment decisions.^15^ The problem is exacerbated at the low radiation doses required for pediatric imaging or frequent follow‐up scans, where increased image noise can render conventional MMD algorithms unstable and unreliable.^16^, ^17^ These limitations underscore the need for a more robust, accurate, and noise‐resilient MMD methodology that can provide reliable quantitative results across a range of patient sizes and clinical scenarios.

While DECT improves material characterization compared to single‐energy CT, iodine quantification accuracy varies across platforms and protocols.^10^, ^18^ Residual errors from dense materials such as calcium remain a major source of inaccuracy, with size‐dependent effects that motivate the need for robust decomposition algorithms.^9^ Accordingly, MMD methods have been central to addressing these residual errors. As dual‐energy measurements alone are insufficient for voxel‐wise three‐material decomposition, the volume conservation principle was introduced as a necessary constraint.^19^ Leveraging this, Mendonça's benchmark barycentric coordinate‐based method (BC‐MMD)^20^ enabled practical three‐material decomposition but often amplified noise. Subsequent strategies attempted to mitigate these limitations with varying success: Harms et al.^21^ proposed penalized weighted least‐squares with similarity‐based regularization (PWLS‐SBR) to reduce noise, though at the risk of material distortion and complex parameter tuning. Lyu et al.^22^ achieved high accuracy using nonconvex sparsity regularization, but with substantial computational cost and manual parameter adjustment, potentially reducing reproducibility. Similarly, statistical methods^23^ and hybrid models (e.g., PWLS‐aviNLM^24^) improved noise modeling yet retained complexity or residual artifacts. Moreover, $Z_{\mathrm{eff}}$‐based MMD approaches^25^ have shown utility in pulmonary perfusion imaging, but their performance remains limited by two‐material assumptions in calcium‐ or lipid‐rich regions. Recent dual‐source PCCT methods^26^ still face calcium‐related residual challenges. Consequently, a fast and accurate method that achieves both low residual error and clinically feasible computation remains an unmet clinical need.

This study introduces and evaluates a principal component analysis multimaterial decomposition (PCA‐MMD) algorithm that performs barycentric decomposition under geometric constraints in a PCA‐transformed feature space. We compared it with the BC‐MMD^20^ using a thorax phantom across patient‐equivalent sizes and radiation dose levels, focusing on iodine quantification accuracy and residual error reduction in the thoracic region relevant to cardiac and lung perfusion imaging.^27^, ^28^ Additionally, the clinical feasibility of the method was also evaluated in a proof‐of‐concept retrospective patient study.

## 2 MATERIALS AND METHODS

### 2.1 Phantom Design and Preparation

A thorax phantom (QRM GmbH, Möhrendorf, Germany) composed of tissue‐equivalent materials (lung: ∼−800 HU, soft tissue: ∼30 HU, bone: ∼200–550 HU) was used as the base, measuring 20×30×10 cm with a water‐equivalent diameter ($D_{w}$) of 20.9 cm. A custom 10‐cm PMMA cylindrical insert with four 2.5‐cm diameter holes was positioned in the cardiac region. Calibrated rod inserts (Sun Nuclear Corporation, Melbourne, FL, USA) included iodine (2, 2.5, 5, 7.5, 10, 15, and 20 mg/mL) and calcium hydroxyapatite (50, 100, 200, 300, and 400 mg/mL) concentrations. Each rod insert contained a single material (either an iodine or a calcium mixture); no combined‐material inserts were used in the phantom study. The four holes were filled interchangeably with three rod‐insert sets: (i) iodine at 2 and 20 mg/mL with calcium at 50 and 400 mg/mL, (ii) iodine at 2.5 and 15 mg/mL with calcium at 100 and 300 mg/mL, and (iii) iodine at 5, 7.5, and 10 mg/mL with calcium at 200 mg/mL, for a total of 12 rod inserts.

To simulate varying patient sizes, two custom fat‐equivalent rings each (5‐cm thick) were 3D‐printed using eSun ABS red filament with ∼100% infill.^29^ These were added to the base phantom to create three adult body‐habitus configurations. The large configuration (40 cm AP × 50 cm LAT) represented a large/bariatric patient. Three phantom configurations were created: small (base phantom, $D_{w}=20.9$ cm), medium (base + one ring, $D_{w}=27.3$ cm), and large (base + two rings, $D_{w}=33.2$ cm). As per AAPM Report 220 guidelines^30^ for heterogeneous phantoms, $D_{w}$ was calculated using an image‐based method, accounting for the low‐density lung‐equivalent material as the appropriate metric for dose characterization. Three phantom sets were scanned with inserts exchanged between acquisitions, yielding 12 unique material configurations.

### 2.2 Scanning protocol

All scans were performed using a cardiac PCCT (NAEOTOM Alpha, Siemens Healthineers, Erlangen, Germany) protocol: 140 kVp with 0.6 mm tin (Sn) filtration, pitch 0.8, collimation 144×0.4 mm. The reference CTDI_vol_ was 54.7 mGy for the large phantom, based on clinical CCTA protocols for patients with $D_{w}>30$ cm.^31^ Tube current was adjusted for smaller phantoms to maintain approximately constant size‐specific dose estimate (SSDE), reflecting clinical dose modulation practices. At the 100% reference dose level, the applied tube currents were 476, 601, and 751 mA for the small, medium, and large phantom configurations, respectively. Each configuration was scanned three times to assess reproducibility.

### 2.3 Dose characterization

Both CTDI_vol_ and SSDE were calculated to comprehensively characterize radiation exposure. CTDI_vol_ represents the standardized dose index for a 32‐cm reference phantom, while SSDE adjusts this value for patient‐specific size using conversion factors from AAPM Report No. 204 and water‐equivalent diameter per AAPM Report No. 220 ^30^, ^32^:

(1) $$\text{SSDE}=\text{CTDIvol}\times f(D_{w})$$

Conversion factors were linearly interpolated from AAPM tabulated values: small phantom: f=1.72 (between $D_{w}=20$ and 21 cm), medium phantom: f=1.36 (between $D_{w}=27$ and 28 cm), and large phantom: f=1.09 (between $D_{w}=33$ and 34 cm). The CTDI_vol_ for the 100% dose level of the large phantom was determined from the default clinical protocol setting,^31^ and the corresponding SSDE was calculated using the parameters in Table 1. This SSDE was then used to determine the CTDI_vol_ for the medium and small phantoms via the corresponding f‐factors. Dose levels ranging from 100 to 5% of the reference clinical dose were used, achieved by proportionally adjusting the tube current‐time product (mAs) from the size‐specific reference values. Table 1 summarizes the resulting dose metrics.

**TABLE 1:** Radiation dose metrics across phantom sizes and dose reduction levels.

			Dose levels				
Phantom	*D*_w_(cm)	f‐factor (AAPM)	100%	50%	25%	10%	5%
			**CTDI_vol_ (mGy)**				
Small	20.9	1.72	34.7	17.4	8.7	3.5	1.7
Medium	27.3	1.36	43.8	21.9	11.0	4.4	2.2
Large	33.2	1.09	54.7	27.4	13.7	5.5	2.7
			**SSDE (mGy)**				
All Sizes	—	—	59.6	29.8	14.9	6.0	2.9

### 2.4 Image reconstruction

Images were reconstructed using Quantum Iterative Reconstruction (QIR) level 3, with kernel Bv64 (vascular‐sharp), matrix size 512×512, and 3‐mm slice thickness. These settings improved spatial resolution and reduced blooming artifacts in PCCT cardiac imaging.^33^ Virtual monoenergetic images (VMIs) at 70 and 150 keV were generated using Syngo.via (Siemens Healthineers). The 70‐keV images provided optimal contrast‐to‐noise ratio for iodine visualization, while 150‐keV images minimized beam hardening for accurate material decomposition.^33^

### 2.5 Multimaterial decomposition methods

#### 2.5.1 BC‐MMD method

Although DECT provides only two measurements per voxel,^34^ BC‐MMD extends decomposition to three materials by enforcing volume preservation constraints.^19^, ^20^

The algorithm assumes materials mix as ideal solutions, where the linear attenuation coefficient is modeled as follows:

(2) $$\mu_{L}\left(E\right)=\sum_{i=1}^{3}\alpha_{i}\mu_{L,i}\left(E\right),\;\text{subject to}\;\sum_{i=1}^{3}\alpha_{i}=1,\;0\leq \alpha_{i}\leq 1$$

with $\alpha_{i}$ representing volume fractions and $\mu_{L,i}\left(E\right)$ the attenuation coefficient of material i at energy E. The volume constraint provides the third equation required for three‐material decomposition from two energy measurements.

**FIGURE 1:**
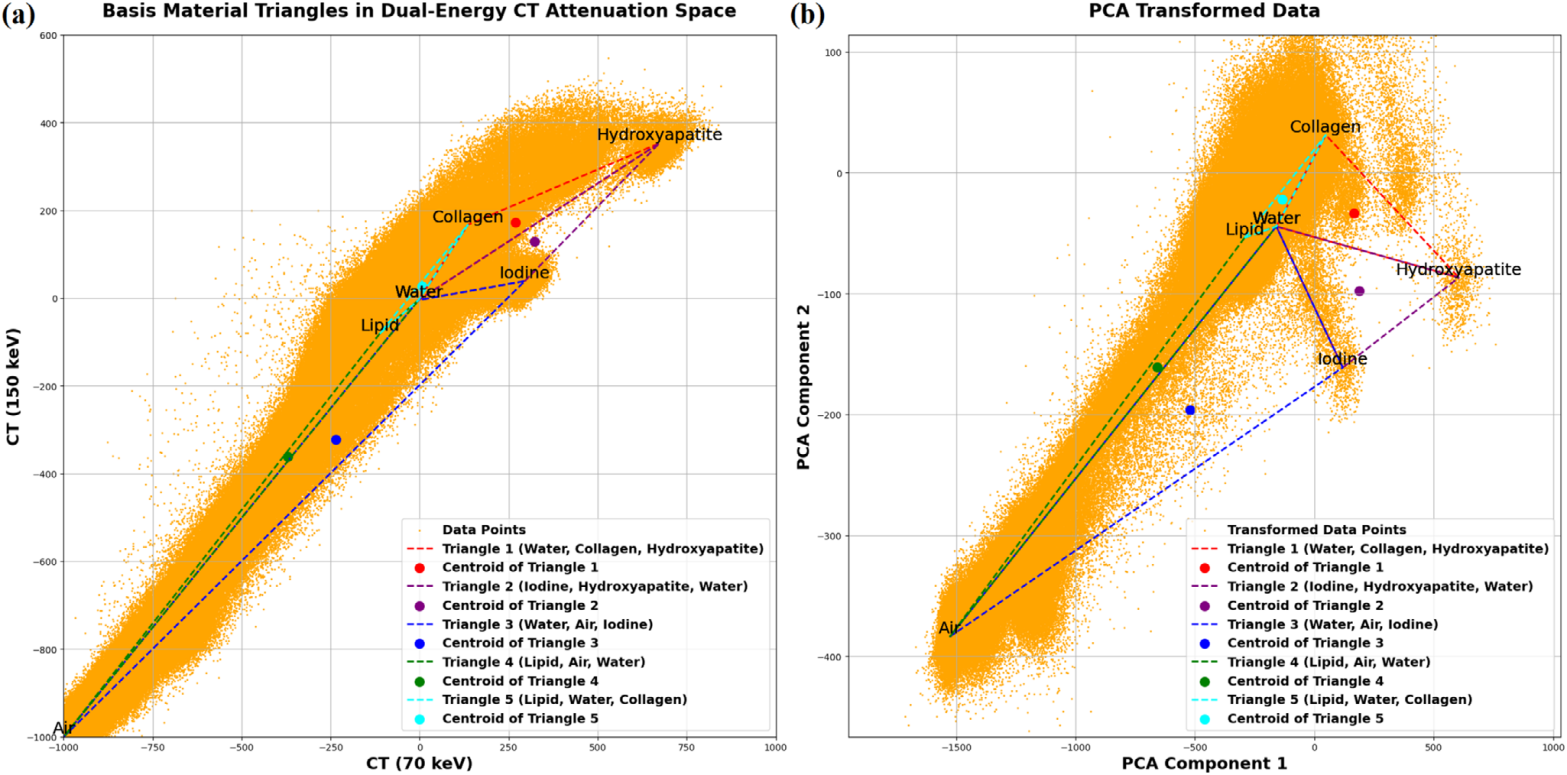
PCA transformation on material separability. (a) Original dual‐energy attenuation space (used in BC‐MMD) showing overlapping material clusters and ambiguous triangle configurations. (b) PCA‐transformed space (used in PCA‐MMD) exhibiting improved material separability with well‐defined geometric boundaries between materials. Centroids (filled circles) indicate the reference points used for triangle selection.

For each voxel with attenuation vector $\mu_{L}=\left(\mu_{L}\left(E_{1}\right),\mu_{H}\left(E_{2}\right)\right)$, the algorithm searches a library of material triplets T, where each triplet defines a triangle in 2D attenuation space (Figure 1a). Decomposition involves solving:

(3) $$\left[\begin{array}{ccc} \mu_{L,1}\left(E_{1}\right) & \mu_{L,2}\left(E_{1}\right) & \mu_{L,3}\left(E_{1}\right)\\ \mu_{H,1}\left(E_{2}\right) & \mu_{H,2}\left(E_{2}\right) & \mu_{H,3}\left(E_{2}\right)\\ 1 & 1 & 1 \end{array}\right]\left[\begin{array}{c} \alpha_{1}\\ \alpha_{2}\\ \alpha_{3} \end{array}\right]=\left[\begin{array}{c} \mu_{L}\left(E_{1}\right)\\ \mu_{H}\left(E_{2}\right)\\ 1 \end{array}\right]$$

A solution is feasible if all $\alpha_{i}\in \left[0,1\right]$, indicating μ lies within the triangle. The algorithm selects the first triplet yielding a feasible solution. When no feasible solution exists—typically due to noise or unmodeled materials—it selects the triplet minimizing the Hausdorff distance, which may yield fractions outside [0,1], indicating unphysical solutions.

#### 2.5.2 PCA‐MMD method

To address the limitations of the BC‐MMD algorithm, we propose the PCA‐MMD algorithm, which introduces three key enhancements while preserving the deterministic, parameter‐free nature of the original approach.

The first enhancement addresses the complex patterns and noise inherent in dual‐energy attenuation data. Scanner characteristics, beam hardening effects, and anatomical heterogeneity create measurement distributions that challenge direct geometric analysis in the original $\left(\mu_{L},\mu_{H}\right)$ space (Figure 1a). We therefore transform the data using Principal Component Analysis (PCA), which rotates the measurement space to align with the principal axes of variance (Figure 1b). VMIs at 70 and 150 keV were generated from the PCCT scanner using the Syngo.via platform (Siemens Healthineers). These VMIs served as input for all decomposition algorithms, providing dual‐energy datasets with dimensions S×H×W (where S is the number of slices and H×W are the in‐plane dimensions). Each voxel yields a paired measurement $X\in R^{N\times 2}$, where N=S×H×W denotes the total number of voxels. The ith row of X contains $\left[\mu_{L}\left(x_{i}\right),\mu_{H}\left(x_{i}\right)\right]$, corresponding to the low‐ and high‐energy attenuation values at spatial position $x_{i}$. We first compute the mean‐centered data:

(4) $$\tilde{X}=X-1{\bar{\mu }}^{T},\;\text{where}\;\bar{\mu }=\frac{1}{N}\sum_{i=1}^{N}\left[\begin{array}{c} \mu_{L}\left(x_{i}\right)\\ \mu_{H}\left(x_{i}\right) \end{array}\right]$$

where $1\in R^{N}$ is a vector of ones. The covariance matrix and its eigenvalue decomposition yield:

(5) $$C=\frac{1}{N-1}{\tilde{X}}^{T}\tilde{X}=W\Lambda W^{T}$$

where $W=[w_{1},w_{2}]$ contains eigenvectors and diagonal matrix $\Lambda =\text{diag}(\lambda_{1},\lambda_{2})$ contains eigenvalues, which represent the variance along each principal component direction. We then project all voxel measurements and material library entries into PCA space:

(6) $$\mu^{\prime }\left(x\right)=W^{T}\left[\mu \left(x\right)-\bar{\mu }\right],\;m_{m}^{\prime }=W^{T}\left[m_{m}-\bar{\mu }\right]$$

where $\mu^{\prime }\left(x\right)={\left[\mu_{1}^{\prime }\left(x\right),\mu_{2}^{\prime }\left(x\right)\right]}^{T}$ denotes the transformed attenuation vector in PCA space, and $m_{m}={\left[\mu_{L,m},\mu_{H,m}\right]}^{T}$ represents the attenuation vector of material m at the low and high energies obtained from the NIST database.^35^ Our six‐material library (air, water, iodine, hydroxyapatite, collagen, lipid) forms five triangles in PCA space (Figure 1b), each defined by transformed vertices $T_{k}^{\prime }=m_{i}^{\prime },m_{j}^{\prime },m_{k}^{\prime }$. These triangles exhibit better separation and reduced overlap than in the original space.

Instead of solving potentially ill‐conditioned linear systems, we compute barycentric coordinates using signed triangle areas. For a triangle with vertices $v_{0}^{\prime },v_{1}^{\prime },v_{2}^{\prime }$ and a point $p^{\prime }$, the barycentric coordinates are as follows:

(7) $$\lambda_{i}=\frac{A(p^{\prime },v_{j}^{\prime },v_{k}^{\prime })}{A(v_{0}^{\prime },v_{1}^{\prime },v_{2}^{\prime })}$$

where (i,j,k) are cyclic permutations of (0,1,2) and the signed area is as follows:

(8) $$A\left(a,b,c\right)=\frac{1}{2}\left|\begin{array}{ccc} a_{x} & a_{y} & 1\\ b_{x} & b_{y} & 1\\ c_{x} & c_{y} & 1 \end{array}\right|$$

This area‐based formulation avoids the instability of matrix inversion and guarantees robust computation of $\lambda_{i}$.

To assign each voxel point to the most appropriate triangle in PCA space, we define a centroid distance metric ($d_{k}$) that measures the Euclidean distance from a point to a triangle's centroid, facilitating consistent triangle selection across various geometric configurations. Formally, the centroid distance metric is defined as follows:

(9) $$d_{k}={∥p^{\prime }-c_{k}^{\prime }∥}_{2},\;\text{where}\;c_{k}^{\prime }=\frac{1}{3}\sum_{i=0}^{2}v_{i}^{\prime }$$

This metric measures how close a voxel point is to the triangle's centroid (mean of vertex coordinates) and serves as a consistent criterion for selecting the most representative triangle in material decomposition. It is then applied consistently across the three‐pass geometric strategy to ensure robust and physically meaningful triangle assignments. Rather than the priority‐based search used by BC‐MMD, PCA‐MMD employs a geometrically motivated three‐pass strategy. For each PCA‐transformed voxel point $p^{\prime }=\mu^{\prime }\left(x\right)$:

*Pass 1 —Interior point detection*: We test if $p^{\prime }$ lies within any triangle using Equation (7). A point is inside when all $\lambda_{i}\in \left[-\varepsilon ,1+\varepsilon \right]$ with $\varepsilon =10^{-6}$ and $\sum_{i}\lambda_{i}\approx 1$. If multiple triangles contain $p^{\prime }$, we select the triangle with the smallest centroid distance $d_{k}$ to the voxel point (Equation 9).

*Pass 2 —Edge‐adhering candidate triangles*: If $p^{\prime }$ does not fall within any triangle interior, we evaluate whether it lies on any triangle edge (within numerical tolerance), which often corresponds to partial volume effects at material boundaries. For each edge defined by vertices $\left(v_{a}^{\prime },v_{b}^{\prime }\right)$, we compute the perpendicular distance:

(10) $$d=\min_{t\in [0,1]}{\left\|p^{\prime }-\left[\left(1-t\right)v_{a}^{\prime }+tv_{b}^{\prime }\right]\right\|}_{2}$$

If d<ε, the point is considered to lie on the edge. Among triangles with such adhering edges, we select the one with the smallest centroid distance $d_{k}$ (Equation 9). This ensures consistent handling of boundary cases, favoring the triangle that best represents the local material mixture and minimizing bias from arbitrary ordering.

*Pass 3 —Constrained geometric projection*: For voxels that remain unassigned (i.e., points outside all triangle interiors and not adhering to any edges, typically due to noise or measurement outliers), we project $p^{\prime }$ onto the nearest edge across all triangles to enforce a physically meaningful assignment:

(11) $$p_{\text{proj}}^{\prime }=\arg\min_{q\in {⋃}_{k}\partial T_{k}^{\prime }}{∥p^{\prime }-q∥}_{2}$$

where $\partial T_{k}^{\prime }$ denotes the set of edges forming triangle $T_{k}^{\prime }$. We select the triangle containing the edge with the minimal projection distance. In the rare event of ties (multiple edges at the exact minimal distance), we select the triangle with the smallest centroid distance $d_{k}$ (Equation 9), maintaining the consistency of our selection criterion across all passes. Barycentric coordinates are then recomputed using $p_{\text{proj}}^{\prime }$, ensuring non‐negative, bounded material fractions that sum to one—even in the presence of high noise levels or outlier conditions.

Finally, with the appropriate $p_{\text{proj}}^{\prime }$, the resulting $\lambda_{i}$ values obtained via Equation (7) directly represent material volume fractions. This three‐pass geometric framework ensures that every voxel is assigned physically valid fractions ($\lambda_{i}\in \left[0,1\right]$, $\sum_{i}\lambda_{i}=1$), thereby overcoming the instability, bias, and nonphysical outputs associated with BC‐MMD.

### 2.6 Data analysis

All statistical analyses and visualizations were performed using Python (version 3.12) with libraries including NumPy, SciPy, Matplotlib, ipywidgets, and IPython.display. Data from PCCT images at 70 and 150 keV were processed voxel‐wise to generate volumetric fraction maps for six basis materials (air, water, lipid, iodine, collagen, and hydroxyapatite). For the iodine basis material, we used the elemental composition of the 20‐mg/mL Sun Nuclear iodine rod from,^36^ with theoretical attenuation coefficients calculated from the NIST database.^37^ Because the algorithm uses the exact rod composition as triangle vertices, the volumetric fractions directly represent the proportion of each voxel corresponding to that rod. Iodine concentrations were then obtained by the following:

(12) $$C_{\text{iodine}}=f_{\text{iodine}}\times 20\;\text{mg/mL}$$

where $f_{\text{iodine}}$ is the volumetric fraction (0–1) relative to the 20‐mg/mL rod composition. This approach requires no additional calibration step since the basis materials match the phantom rods exactly. The conversion is valid assuming: (i) the reported elemental compositions accurately represent the Sun Nuclear rods, and (ii) the fraction–concentration relationship remains linear with zero intercept across the 0–20‐mg/mL range, as mentioned by Yu et al.^38^ Subsequent quantitative evaluations focused on these iodine concentration maps and noniodine residuals in the iodine map.

Iodine quantification accuracy was evaluated by comparing estimated concentrations against known reference values (2, 2.5, 5, 7.5, 10, 15, and 20 mg/mL) in phantom inserts. Weighted linear regression was applied to unity line plots using measurement uncertainties as weights, fitting the model $C_{\text{estimated}}=mC_{\text{reference}}+c$, where m and c denote the slope and intercept. The coefficient of determination ($R^{2}$) was computed as ($R^{2}=1-\sum {\left(C_{\text{estimated},i}-{\hat{C}}_{i}\right)}^{2}/\sum {\left(C_{\text{estimated},i}-{\bar{C}}_{\text{estimated}}\right)}^{2}$). Root mean square error (RMSE) was calculated as follows:

(13) $$\text{RMSE}=\sqrt{\frac{1}{n}\sum_{i=1}^{n}{\left(C_{\text{estimated},i}-C_{\text{expected},i}\right)}^{2}}$$

where n is the number of measurements, $C_{\text{estimated},i}$ is the estimated concentration, and $C_{\text{expected},i}$ is the expected value (2–20 mg/mL for iodine ROIs, and 0 mg/mL for noniodine ROIs). RMSE was used to assess the accuracy of iodine quantification across methods, phantom sizes, and dose levels (3–54 mGy).

For noniodine ROIs (calcium inserts and lung regions), the expected iodine concentration is 0 mg/mL. Any nonzero value indicates a false‐positive iodine signal. To express the magnitude of this error relative to the method's sensitivity, we define the residual error percentage as follows:

(14) $$\text{Residual error}\left(\%\right)=\frac{{\bar{C}}_{\text{non-iodine}}}{C_{\min}}\times 100$$

where ${\bar{C}}_{\text{non-iodine}}$ is the combined mean iodine concentration across all calcium and lung ROIs, and $C_{\min}$ is the measured iodine concentration for the 2.0‐mg/mL reference insert specific to each method. This normalization expresses the false‐positive iodine signal in noniodine regions relative to the method‐specific lowest detectable iodine concentration, providing an intuitive measure of the algorithm's ability to suppress residual errors while maintaining sensitivity to low iodine concentrations. ROI placements are illustrated on the iodine map of Set 3 (Section 2.1) in Figure 3.

**FIGURE 2:**
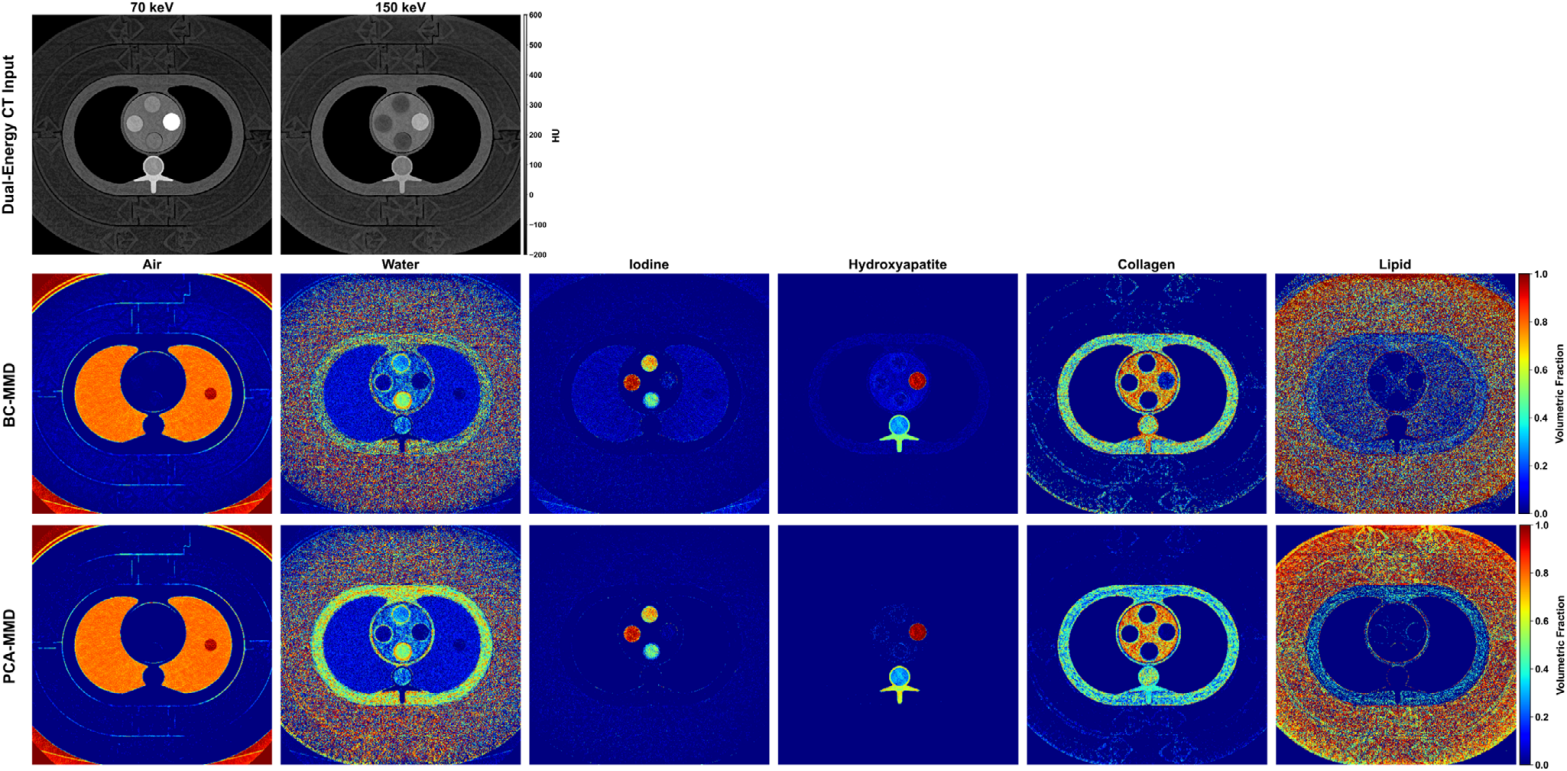
Visualization of material fraction maps for six basis materials (air, water, iodine, hydroxyapatite, collagen, and lipid) obtained from dual‐energy PCCT images reconstructed at 70 and 150 keV. Top row: input CT images (WW/WL = 400/50 HU). Middle row: BC‐MMD decomposition results. Bottom row: PCA‐MMD decomposition results.

**FIGURE 3:**
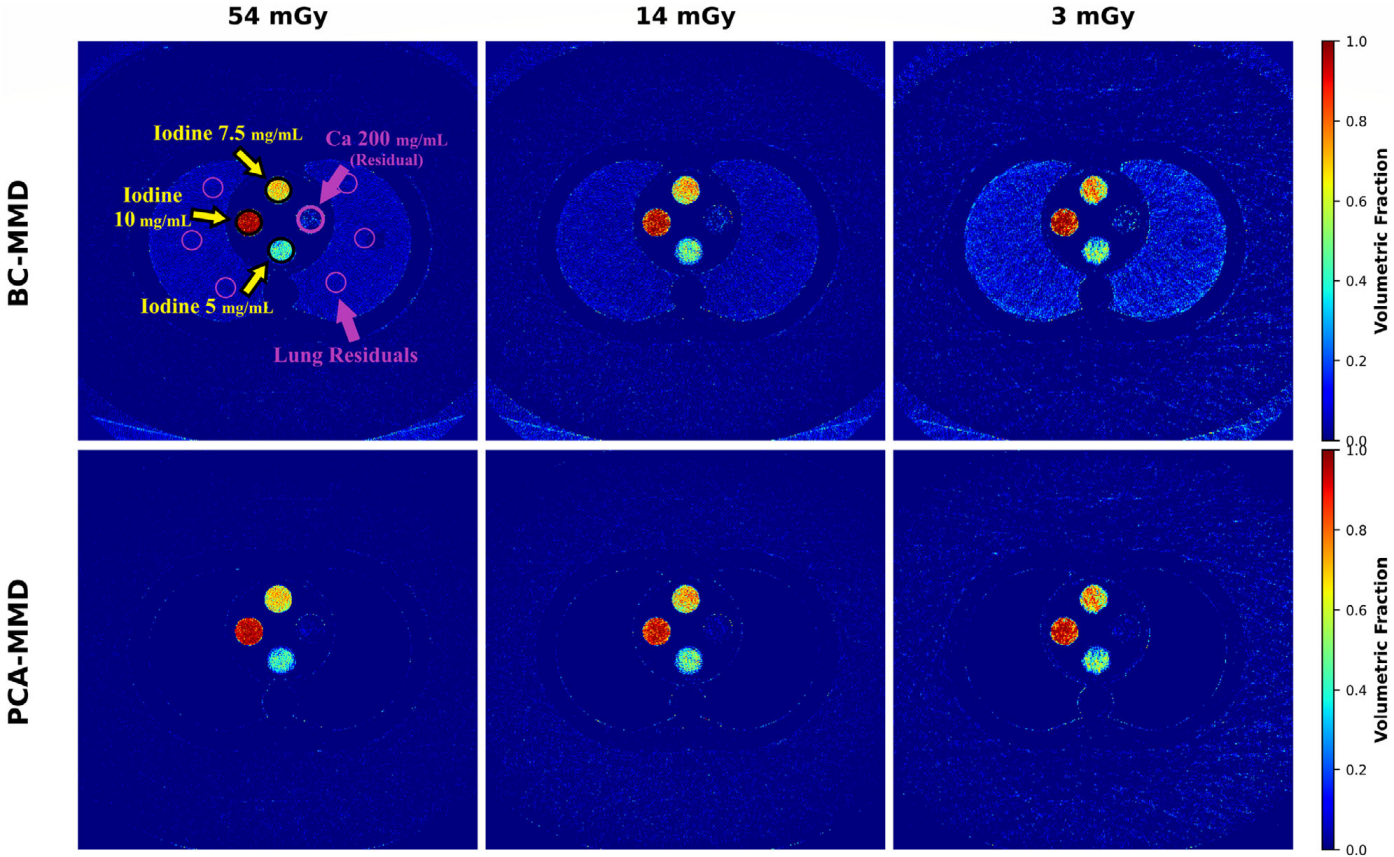
Visualization of iodine material fraction maps across dose levels in the large phantom using BC‐MMD and PCA‐MMD. Set 3 is shown as a representative case due to its intermediate and diverse iodine concentrations, which best highlight the differences between methods. Top row: BC‐MMD results, showing residual errors especially in noniodine regions. Bottom row: PCA‐MMD results, demonstrating stable iodine maps across various dose levels with very low residuals, even at ultra‐low doses.

Reproducibility was assessed by performing three repeated scans at 25% dose and evaluating the consistency of the resulting iodine‐decomposed maps. This dose level was specifically chosen to provide a meaningful stress test for algorithmic stability. At the 100% dose level, the signal‐to‐noise ratio is very high, and most algorithms are expected to perform consistently. Conversely, results at the 5% level are dominated by quantum noise, making it difficult to distinguish algorithmic instability from the inherent noise in the data. Therefore, the 25% level was selected because it represents a clinically relevant intermediate‐noise condition. This allows for a fair assessment of the algorithm's intrinsic consistency, distinct from the effects of extreme noise or near‐perfect acquisition conditions. ROIs were placed directly over the iodine rods in each decomposed map, and the coefficient of variation (CV) was calculated for each rod:

(15) $$\text{CV}\left(\%\right)=\left(\frac{\sigma_{C}}{\bar{C}}\right)\times 100$$

where $\bar{C}$ is the mean iodine concentration measured in a given rod's ROI across the three decomposed maps, and $\sigma_{C}$ is the corresponding standard deviation for that same rod. This rod‐by‐rod analysis quantified the reproducibility of the decomposition algorithm under identical scanning conditions.

CV values were categorized as “Excellent” (<2%), “Good” (2%–5%), or “Acceptable” (5%–10%) to provide interpretive granularity within the generally accepted reproducibility range, where CV <10% is considered adequate for imaging measurements.^39^, ^40^ This subdivision was introduced solely to reflect how reproducibility improves within our method across repeated scans.

### 2.7 Statistical analysis

All statistical analyses were performed using Python (version 3.12) with SciPy (version 1.13.1) and statsmodels (version 0.14.2). A significance level of α=0.05 was used for all statistical tests.

#### Phantom experiments

Iodine quantification accuracy was assessed for each experimental condition (phantom size and dose level) using the seven iodine rods (2–20 mg/mL) described in Section 2.6. $C_{\text{meas}}$ for each rod was computed as the volumetric mean across the z‐extent of the rod ROI, and signed error was defined as $d=C_{\text{meas}}-C_{\text{true}}$. Paired differences in absolute error were computed as $\Delta =|d_{\text{PCA-MMD}}\left|-\right|d_{\text{BC-MMD}}|$, and a two‐sided paired t‐test was used to test whether the mean of Δ differed from zero (i.e., whether PCA‐MMD statistically reduced absolute error magnitude relative to BC‐MMD), treating each iodine insert as the independent unit.

Residual iodine was evaluated in noniodine ROIs (lung‐equivalent regions and calcium inserts) with expected iodine concentration zero, using the volumetric mean false‐positive iodine signal per ROI. Paired differences were computed as $\Delta_{r}=|r_{\text{PCA-MMD}}\left|-\right|r_{\text{BC-MMD}}|$, and a two‐sided paired t‐test was applied (N=11) to test whether PCA‐MMD statistically reduced residual error magnitude relative to BC‐MMD.

#### Patient study

Clinical accuracy was assessed by comparing mean virtual noncontrast (VNC) CT numbers to the paired true noncontrast (TNC) reference using 25 anatomically defined ROIs (five ROIs per subject across five subjects; Section 2.8). Absolute error was computed as $\varepsilon =|HU_{\text{VNC}}-HU_{\text{TNC}}|$. Normality of paired differences between methods was verified using the Shapiro–Wilk test (p>0.05); therefore, a paired two‐sided Student's t‐test was used to compare PCA‐MMD against vendor VNC and BC‐MMD, with p<0.05 considered statistically significant.

### 2.8 Clinical proof‐of‐concept evaluation

As an initial proof‐of‐concept evaluation of the proposed PCA–MMD method, paired TNC and contrast‐enhanced ECG‐gated CCTA datasets from five adult subjects were retrospectively collected under Institutional Review Board (IRB) approval (Protocol #6974, exempt protocol involving de‐identified patient data). All scans were performed on the PCCT system described in Section 2.2. The cohort size is consistent with prior proof‐of‐concept DECT studies, including Li et al.^25^ Subject demographics and scan parameters are summarized in Table 2. Each subject underwent a TNC chest scan and a contrast‐enhanced CCTA acquisition reconstructed at the vendor‐selected best diastolic phase (∼75% RR). Unlike phantom experiments, ground truth material composition is not available in vivo; thus TNC images serve as the closest available clinical reference for evaluating virtual noncontrast (VNC) images generated by MMD methods. Accordingly, we employed a paired TNC–VNC comparison strategy, following the standard clinical validation framework established in previous studies.^20^, ^41^, ^42^ Low‐ and high‐energy VMIs were generated from CCTA using Syngo.via (Siemens Healthineers) and processed by each MMD method to produce material maps. VNC images were generated by subtracting the decomposed iodine component from the contrast‐enhanced VMI.^43^, ^44^, ^45^, ^46^ To ensure voxel‐wise correspondence between VNC and TNC images, rigid ANTs (advanced normalization tools) registration was applied to achieve consistent image geometry (field‐of‐view, pixel spacing, and slice thickness). Quantitative evaluation was performed using anatomically defined ROIs derived from automated segmentation masks (TotalSegmentator^47^) for the aorta, atrial blood pools, and ventricular blood pools, minimizing user bias and improving reproducibility. Summary metrics, including RMSE and RE, were calculated within these ROIs.

**TABLE 2:** Subject demographics and retrospective scan and reconstruction parameters for the clinical proof‐of‐concept evaluation.

Parameter	P1	P2	P3	P4	P5
*Demographics*					
Age (years)	91	67	64	66	67
Sex	M	F	M	F	F
Weight (kg)	78.4	73.3	73.5	130.3	71.8
Water‐equivalent diameter, $D_{w}$ (cm)	27.5	33.4	31.1	28.2	25.4
*Acquisition parameters*					
Scanner	Siemens NAEOTOM Alpha (PCCT)				
Tube potential	140 kVp (Quantum Plus)				
ECG phase	Best diastolic phase (∼75% RR)				
*Reconstruction parameters*					
Reconstruction kernel (TNC)	Qr36f				
Slice thickness (TNC)	2.0 mm				
Reconstruction kernel (CCTA)	Qr40f				
Slice thickness (CCTA)	0.4 mm	0.4 mm	0.4 mm	0.8 mm	0.8 mm

### 2.9 Speed performance measurement

The PCA‐MMD algorithm, a new CUDA‐accelerated^48^ method, exploits voxel‐wise parallelism for PCA projection, triangle selection, and barycentric computation via CUDA kernels. Experiments were conducted on a Dell Precision 3650 workstation (Intel Core i9‐11900 @ 2.50GHz, 8 cores/16 threads, 64 GB RAM) with an NVIDIA GeForce RTX 3060 GPU (3584 CUDA cores). PCA‐MMD was implemented with a 1D grid configuration (256 threads/block, 46 000 blocks), using device functions for signed areas, edge adherence checks, and projections. Precomputed triangle vertices and PCA matrices reside in constant memory, with local arrays for temporary computations. The BC‐MMD method was tested in both single‐threaded and multithreaded (16 threads) CPU implementations. Processing times were measured for 45 slices (512 × 512 pixels, 3‐mm thickness) of the QRM Thorax Phantom, totaling 11.8M voxels.

## 3 RESULTS

### 3.1 Phantom study

Figure 2 compares six‐material decomposition results from PCA‐MMD and BC‐MMD methods.^20^ Both methods produced plausible fraction maps across the full library, including lipid, air (lung), collagen (muscle‐like regions), calcium (bone/rod inserts), and water. Both approaches identified the iodine rods; however, BC‐MMD exhibited strong false‐positive iodine signals in noniodine regions, whereas PCA‐MMD effectively suppressed these residual artifacts. Therefore, the remainder of this section focuses on iodine quantification accuracy and residual suppression as the primary performance benchmarks.

Figure 3 shows iodine fraction maps at 3, 14, and 54 mGy for the large phantom, representing the most challenging scenario. True iodine signal should be confined to the central rod inserts (yellow arrows). However, BC‐MMD produced substantial false‐positive residuals, particularly in the calcium insert and lung regions, which worsened at lower dose. In contrast, PCA‐MMD remained stable across the dose range with markedly reduced residual artifacts. BC‐MMD also generated unphysical values outside [0,1] (min –0.73, max 1.72), whereas PCA‐MMD maintained physically valid fractions.

**FIGURE 4:**
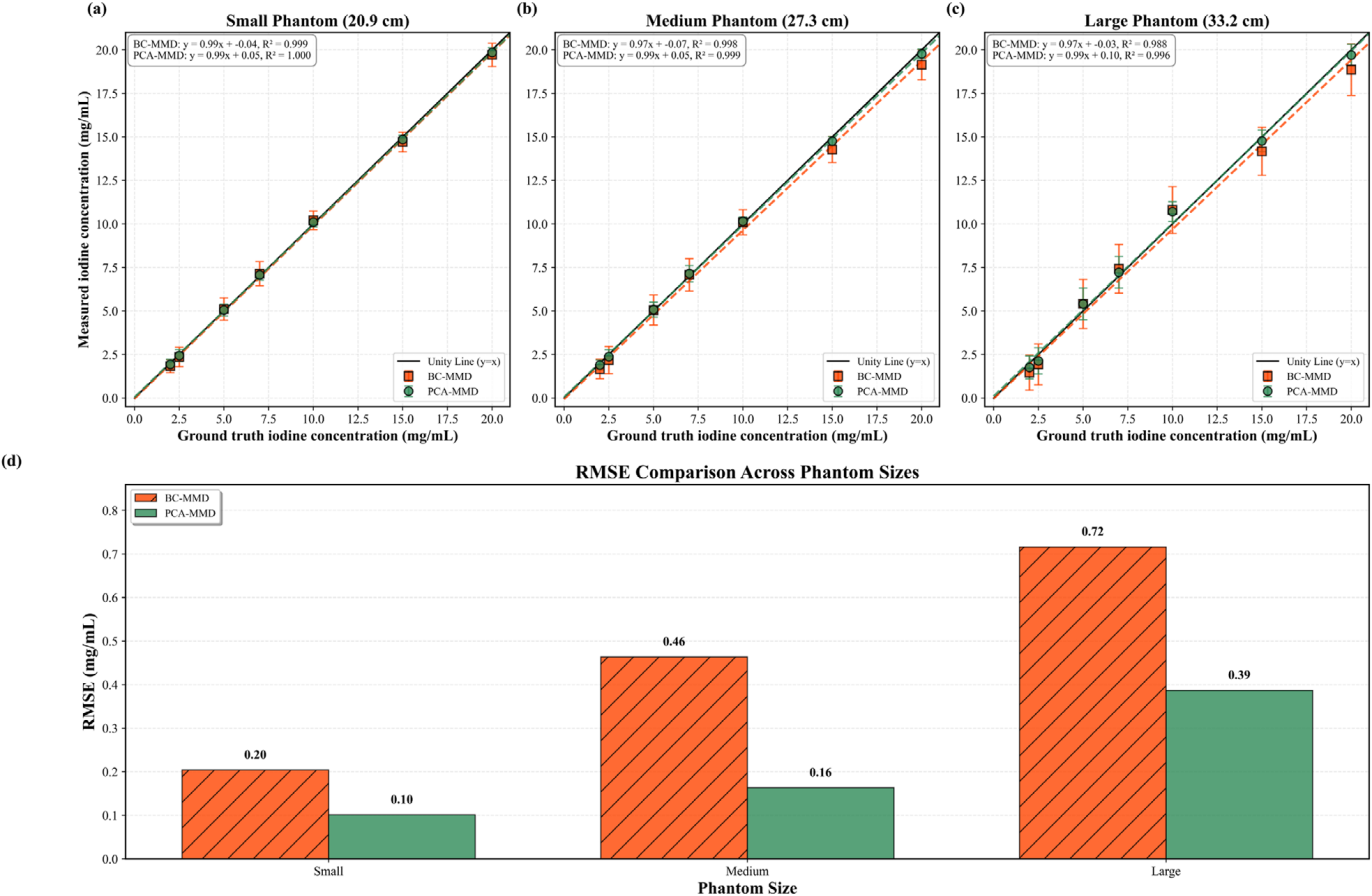
Comparison of iodine quantification accuracy using the BC‐MMD method and the proposed PCA‐MMD method across three phantom sizes (small: 20.9 cm, medium: 27.3 cm, large: 33.2 cm) at 25% dose level (Table 1). (a–c) Unity line plots with linear regression fits and *R* 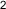 values for both methods. (d) Root mean square error (RMSE) comparison showing PCA‐MMD's improved accuracy, particularly for larger phantoms.

**FIGURE 5:**
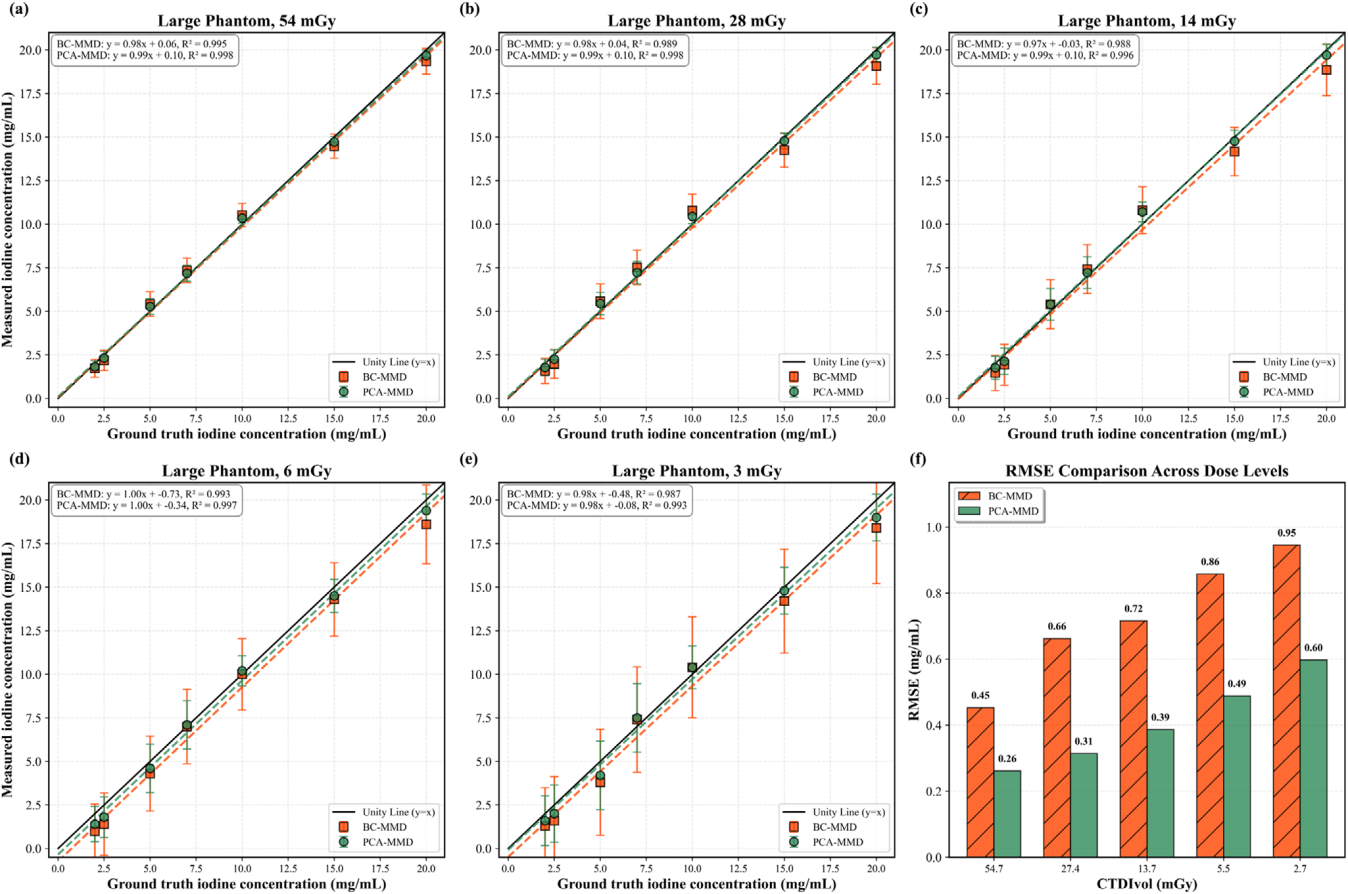
Comparison of iodine concentration estimation across five dose levels (54 to 3 mGy) for a large phantom using the BC‐MMD method and PCA‐MMD. (a–e) Unity line plots with linear fits and *R* 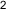 values for both methods. (f) Root mean square error (RMSE) comparison showing PCA‐MMD's superior performance, especially at low doses (e.g., 0.60 vs. 0.95 mg/mL at 3 mGy).

**FIGURE 6:**
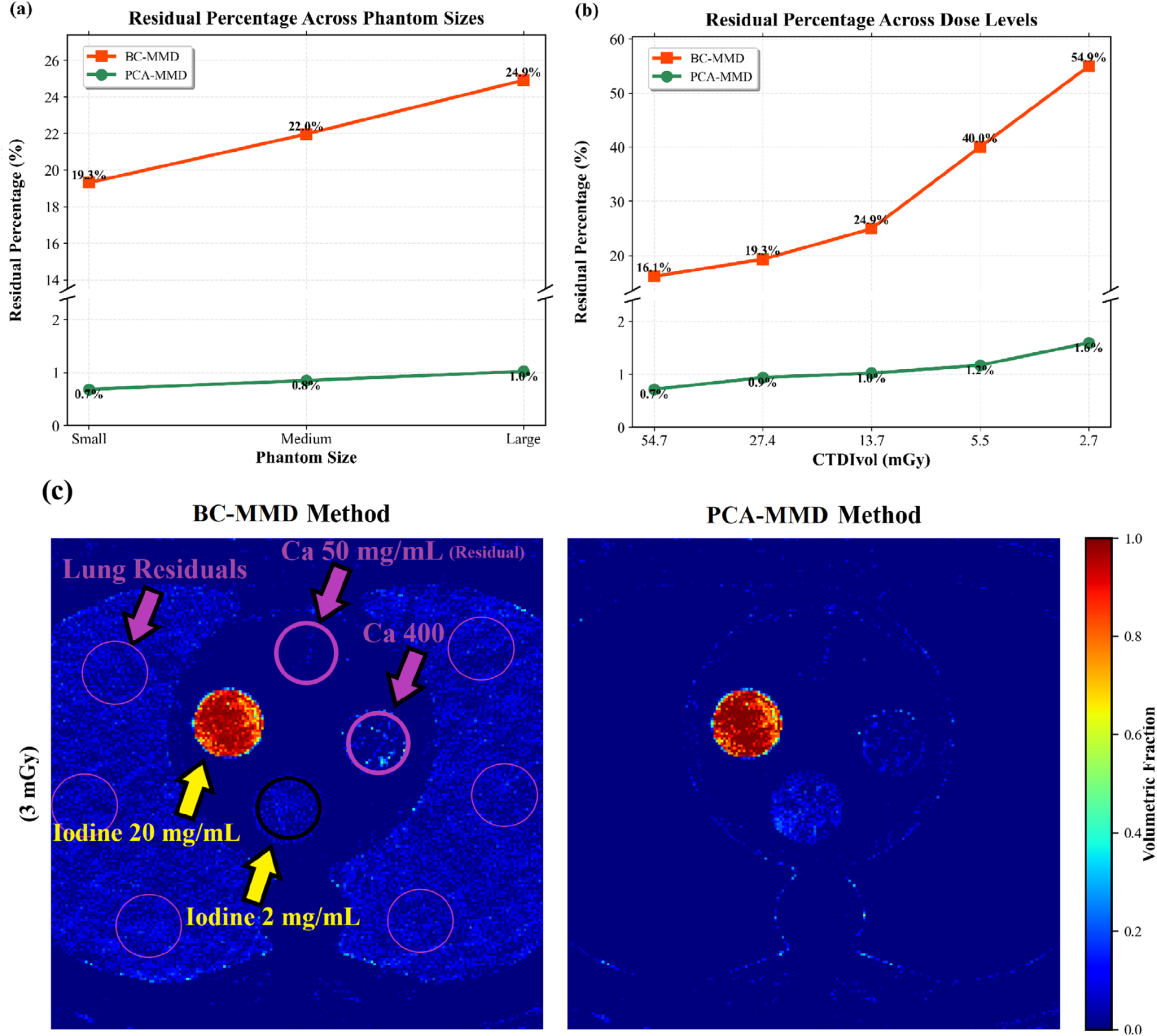
Residual error analysis comparing BC‐MMD and the proposed PCA‐MMD method. (a) Across phantom sizes at the 25% dose level, PCA‐MMD consistently keeps residual errors below 1.0%. (b) Across dose levels for the large phantom, PCA‐MMD remains stable (<1.7%) despite increased noise. (c) Zoomed view of the most challenging case (largest phantom, lowest dose). Set 1 is shown because it contains the lowest iodine concentration (2 mg/mL), where residual signals from BC‐MMD can approach the magnitude of true iodine and risk misinterpretation. In contrast, PCA‐MMD effectively suppresses these false‐positive signals.

The quantitative accuracy of iodine across various phantom sizes is shown in Figure 4. PCA‐MMD achieved regression slopes of 0.99 ($R^{2}=1.000$) for small phantom, 0.99 ($R^{2}=0.999$) for medium, and 0.98 ($R^{2}=0.996$) for large phantom. Regression analysis confirmed excellent linearity for the proposed method across the measured concentration range (p<0.001). Compared with BC‐MMD, PCA‐MMD reduced iodine quantification error by at least a factor of two (Figure 4d). Paired two‐sided t‐tests confirmed statistically significant reductions in absolute error across all phantom sizes (p≤0.029). Reproducibility analysis yielded CV values of 0.51%–4.76% (mean 1.9%), with 85% of measurements rated as excellent (<2%). Dose‐dependent performance is shown in Figure 5. As expected, iodine quantification error increased with decreasing dose for both methods; however, PCA‐MMD demonstrated more consistent linearity and accuracy across dose levels. At the lowest dose (3 mGy), PCA‐MMD maintained $R^{2}=0.993$ with an RMSE of 0.60 mg/mL, compared to BC‐MMD's 0.95 mg/mL, representing a statistically significant improvement (p=0.023). Figure 6 presents the residual errors, computed using Equation (14), in the iodine maps measured from the calcium inserts and lung ROIs. These values reflect residual errors in the decomposition process. PCA‐MMD substantially reduces residual errors across phantom sizes and dose levels compared with the BC‐MMD method, maintaining residual error percentage values below 1.0% (in Figure 6a) at the 25% dose level, and below 1.7% even for the large phantom under decreasing dose levels (in Figure 6b). In the most challenging case (largest phantom at the lowest dose), qualitative inspection (in Figure 6c) reveals that the magnitude of the artifactual residual errors from the BC‐MMD method, especially in the lung region, can approach the true signal intensity of low‐concentration iodine, increasing the risk of false‐positive interpretations, while PCA‐MMD effectively suppresses these false‐positive signals. This reduction was statistically significant (p<0.001).

It should be noted that all three rod‐insert sets were included in the quantitative analysis (regression, RMSE, and residual plots). However, for qualitative visualization, different sets were selected based on purpose. Set 1 was shown in Figure 6 because its lowest iodine concentration (2 mg/mL) makes BC‐MMD residual signals comparable to true iodine, illustrating misinterpretation risk. For the dose‐dependent large‐phantom comparison (Figure 3), Set 3 was selected for brevity, as it spans an intermediate range, includes closely spaced iodine levels (5, 7.5, 10 mg/mL) highlighting sensitivity, provides clearer separation, and contains a 200 mg/mL calcium insert with attenuation within the iodine range, exposing false‐positive artifacts. Set 2 was omitted to avoid redundancy, as it yielded intermediate results without new insights.

Table 3 summarizes the improvements of PCA‐MMD over BC‐MMD, including RMSE reductions of 37%–65% and residual error reductions of 95%–97%. PCA‐MMD was implemented natively on CUDA and enabled full‐volume processing (11.8M voxels) in 0.44 s, corresponding to 3136–6954× speedup relative to CPU‐based BC‐MMD.

**TABLE 3:** Summary of quantitative performance comparing BC‐MMD and PCA‐MMD.

Metric	Condition	BC‐MMD^20^	PCA‐MMD	Error reduction (%)^a^
RMSE (mg/mL)	Small phantom	0.20	0.10	50%
	Medium phantom	0.46	0.16	65%
	Large phantom	0.72	0.39	46%
	3‐mGy dose	0.95	0.60	37%
Residual error (%)	Small phantom	19.3	0.7	96%
	Medium phantom	22.0	0.8	96%
	Large phantom	24.9	1.0	95%
	3‐mGy dose	54.9	1.6	97%
				**Speedup** (×)^b^
Processing time	11.8M voxels	3060 s^c^	0.44 s	6954×
		1380 s^d^	0.44 s	3136×

*Note*: Reported metrics include RMSE, residual percentage, and processing time. The rightmost column shows relative error reduction or speedup.

a Error reduction (%) $=100\times \left(1-\frac{\text{PCA-MMD}}{\text{BC-MMD}}\right)$.

b Speedup (×) $=\frac{\text{BC-MMD time}}{\text{PCA-MMD time}}$.

c Single‐threaded CPU.

d 16‐thread CPU.

* Statistically significant differences in quantification accuracy and residual error were observed across various cases (p<0.05).

### 3.2 Clinical patient study

**FIGURE 7:**
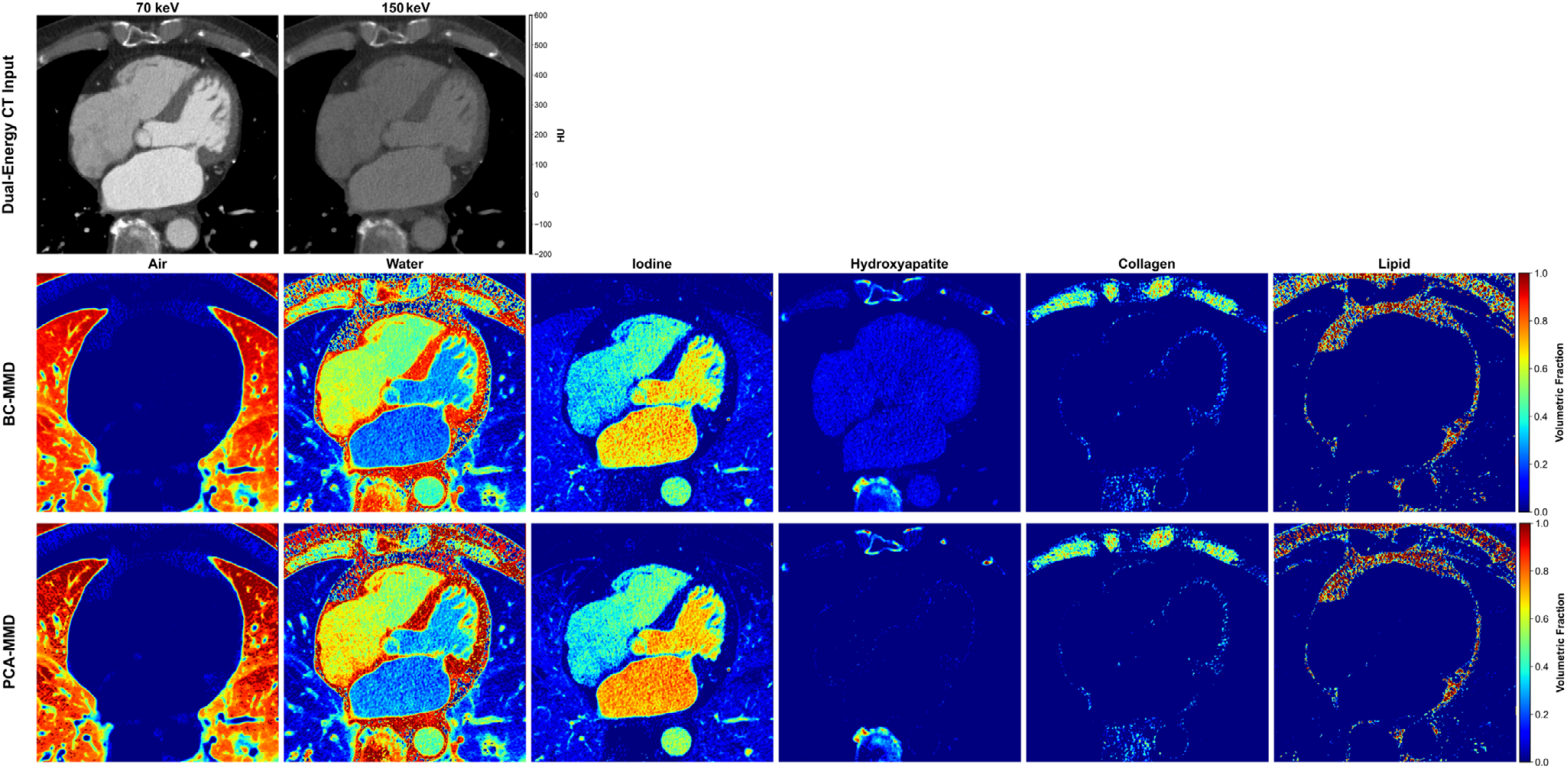
Representative patient results showing six‐material decomposition (air, water, iodine, hydroxyapatite, collagen, and lipid) obtained from dual‐energy CT images reconstructed at 70 and 150 keV. Top row: Input virtual monoenergetic images (WW/WL = 400/50 HU). Middle row: Baseline BC‐MMD decomposition results. Bottom row: Proposed PCA‐MMD decomposition results. Compared with the baseline, PCA‐MMD demonstrates improved confinement of the iodine signal to contrast‐enhanced heart substructures and reduced cross‐talk into noniodine materials, yielding more spatially coherent and anatomically plausible material fraction maps.

**FIGURE 8:**
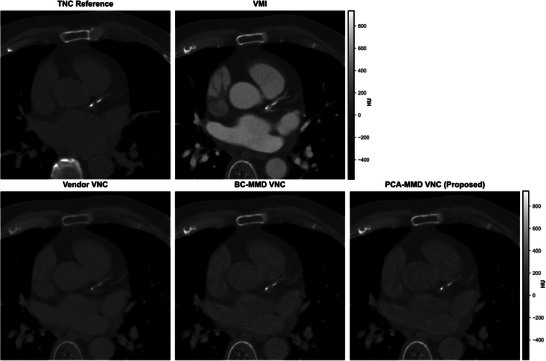
Representative patient results showing the generated virtual noncontrast (VNC) images for the clinical proof‐of‐concept evaluation. The first row displays the true noncontrast (TNC) reference image and the contrast‐enhanced virtual monoenergetic image (VMI) reconstructed at 70 keV. The second row compares VNC images generated using, from left to right, the standard commercial vendor‐specific algorithm, the baseline BC‐MMD method, and the proposed PCA‐MMD approach. Compared to the vendor and BC‐MMD methods, PCA‐MMD demonstrates improved suppression of residual iodine within cardiac substructures and produces a visual appearance that most closely approximates the TNC reference.

Figure 7 presents representative six‐material decomposition results from the clinical cohort. Both BC‐MMD and PCA‐MMD produced material maps consistent with gross anatomy. However, the BC‐MMD iodine map showed false‐positive signals in noncontrast‐enhanced soft tissues and near osseous structures. In contrast, PCA‐MMD yielded a more confined iodine signal within cardiac structures and reduced artifactual noise in the lungs, demonstrating clearer separation of iodine from background soft tissues. Figure 8 shows that the vendor algorithm and BC‐MMD retained residual iodine within cardiac structures, whereas PCA‐MMD achieved more spatially uniform iodine suppression, producing VNC images that more closely approximated the TNC reference.

Quantitative ROI‐based analysis (Table 4) confirmed these qualitative observations. Across the five‐subject cohort, PCA‐MMD achieved the highest accuracy, reducing the mean RMSE to 15.5 HU, compared to 20.9 HU for the vendor‐specific VNC and 25.4 HU for BC‐MMD. Paired two‐sided t‐tests on ROI‐averaged absolute errors confirmed that PCA‐MMD was significantly closer to the TNC reference, reducing the absolute error by 6.6 HU relative to the vendor‐specific VNC (95% CI: [1.6, 11.7] HU; p=0.012) and by 11.5 HU relative to BC‐MMD (95% CI: [7.4, 15.5] HU; p<0.001). Consistent with these findings, PCA‐MMD also achieved the lowest residual error (28.4%), indicating more effective suppression of false‐positive iodine signal relative to the vendor‐specific VNC (45.2%) and BC‐MMD (56.0%).

**TABLE 4:** Quantitative accuracy of VNC methods averaged across five clinical subjects.

	**Method performance**			**Statistical comparison** (p **‐value**)	
**Metric**	**Vendor VNC**	**BC‐MMD**	**PCA‐MMD**	**vs. Vendor**	**vs. BC‐MMD**
RMSE (HU)	20.9	25.4	**15.5**	0.012	<0.001
Residual Error (%)	45.2	56.0	**28.4**		

*Note*: Values represent the mean across 25 anatomical ROIs. p‐values indicate significant reduction in absolute error compared to competing methods. Statistical significance was assessed using a paired two‐sided *t*‐test on the absolute errors. The p‐values demonstrate that PCA‐MMD is statistically significantly closer to the TNC reference compared to both the standard commercial vendor‐specific algorithm (Vendor VNC) and BC‐MMD.

## 4 DISCUSSION

Material decomposition enables the identification and quantification of iodine, which is critical for lesion detection and functional imaging. However, current methods remain limited by iodine quantification accuracy and residual errors in the iodine map, particularly under low‐dose conditions. Here, we evaluated PCA‐MMD, a PCA‐enhanced geometric MMD framework, and demonstrated improved iodine accuracy and reduced residual errors across multiple phantom sizes and dose levels.

Over the past decade, multiple MMD strategies have been proposed. Iterative methods^21^, ^22^, ^23^ can achieve high accuracy but are computationally demanding and require parameter tuning, while deep learning approaches^49^, ^50^, ^51^ remain dependent on training data diversity^52^ and often face generalizability challenges across scanners and noise levels.^53^, ^54^, ^55^ In contrast, deterministic matrix‐based methods^1^, ^20^, ^26^, ^34^ are widely used due to their reproducibility and parameter‐free nature. Therefore, we selected the foundational BC‐MMD method^20^ as our benchmark. Many state‐of‐the‐art iterative pipelines are effectively hybrid systems that pair a baseline decomposition with separate regularization modules (e.g., PWLS‐SBR,^21^ nonlocal means,^24^ total variation,^56^ prior‐image‐based regularization,^57^ or statistical priors^23^, ^58^) making it difficult to attribute performance gains specifically to the decomposition step. This benchmark comparison helps isolate the contribution of the proposed decomposition framework from that of additional regularization and data‐driven modeling components. Accordingly, we position PCA‐MMD as a transparent and hardware‐agnostic enhancement of the core decomposition process, while recognizing direct comparison with DL‐based methods and hybrid integration strategies as important future directions.^59^, ^60^ While BC‐MMD provides a critical benchmark, it can yield (1) unphysical volume fractions (negative or >1), (2) systematic bias from priority‐based triplet selection, and (3) instability near triangle boundaries under noise, particularly in low‐dose imaging. To address these limitations, we propose PCA‐MMD, combining constrained geometric projection and direct area calculations with PCA.

Prior PCA‐based approaches in spectral CT primarily aimed at noise suppression.^61^, ^62^ In contrast, our method not only benefits from PCA's implicit denoising but also leverages variance‐driven feature separation to improve geometric decomposition and material separability. These gains are supported by lower RMSE and residual errors across all dose levels and phantom sizes (Table 3) and by qualitative improvements in Figures 3, 4, 5, 6. The demonstrated improvement can be attributed to the geometric reorientation achieved through PCA, which enhances material separability in the decomposition process. In our implementation, only the first two principal components were retained. PCA projection reorients attenuation data along dominant variance directions, reducing intermaterial overlap and improving triangle distinguishability for barycentric decomposition. As shown in Figure 1, PCA yields more distinct material distributions and clearer triangle boundaries than the original dual‐energy space. This enhanced separability is particularly important under low‐dose conditions where noise predominates. Rather than relying on matrix inversion,^1^, ^20^, ^26^, ^34^ we use a stable geometric formulation (Equation 7) that reduces numerical sensitivity.^63^ Voxels are assigned to a single triangle via a hierarchical rule: centroid‐based selection for overlapping candidates (Equation 9) and edge projection for out‐of‐domain points (Equation 11). This strategy enforces physically valid fractions and improves robustness under under low‐dose conditions. A key challenge in MMD is selecting a basis‐material library that adequately represents biological tissues, yet no universal consensus exists.^1^ This limitation arises from DECT physics: Only two independent measurements are available per voxel, and with volume‐fraction preservation, the problem is restricted to at most three basis materials per voxel. To retain broad representability under this constraint, we adopt a piecewise three‐material framework and define a generalized six‐material library (air, lipid, water, collagen, hydroxyapatite, iodine; Figure 1). This choice is supported by Patern‘o et al.,^64^ who showed that lipid, water, collagen, and hydroxyapatite combinations approximate attenuation across many tissues (e.g., breast, liver, kidney, muscle, and bone). We extend this basis by adding air (lung/body cavities) and iodine (contrast enhancement). Rather than performing an ill‐posed six‐material voxel‐wise decomposition, we partition the attenuation space into five physiologically motivated three‐material domains (triangles in Figure 1). Within this piecewise formulation, each voxel is assigned to a single domain based on its low‐ and high‐energy attenuation values, and only the three materials defining that domain are allowed to assume nonzero volume fractions. In this study, we adopt a default configuration consisting of five three‐material domains designed to capture the most common physiological interfaces: (1) air–soft tissue, (2) fat–muscle, (3) bone–soft tissue, (4) iodine near air, and (5) calcified tissues with iodine. Nonphysiological combinations (e.g., air–iodine–hydroxyapatite) were excluded for interpretability and stability. Overlapping domains are resolved using a deterministic centroid‐based multipass selection rule (Pass 1, Section 2.5.2). Decompositions beyond three materials per voxel generally require additional constraints or a priori information (e.g., histology or ex vivo reference measurements)^65^; in contrast, PCA‐MMD is purely image‐based and suitable for in vivo use. Voxels outside all predefined triangles (noise/partial volume/complex mixtures) are handled via constrained geometric projection to the most appropriate three‐material domain, ensuring numerical stability and physical plausibility. In the following paragraphs, we further discuss key findings and comparisons with previous studies.

The primary finding is that PCA‐MMD achieves up to a 65% relative reduction in iodine quantification error (RMSE) compared to the Mendonça method (Figures 3, 4, 5; Table 3). These improvements were supported by statistical analysis across phantom sizes and dose levels (p<0.05). Notably, PCA‐MMD achieves comparable accuracy at nearly an order of magnitude lower radiation dose. At 3 mGy, PCA‐MMD reaches a similar accuracy level as BC‐MMD at 28 mGy (Figure 5f), which is particularly relevant for dose‐sensitive applications such as pediatric imaging, multiphase perfusion studies, and screening protocols. Residual errors shown in Figures 3 and 6, and Table 3 showed marked differences between methods. For the large phantom at 3 mGy, BC‐MMD produced a 54.9% residual, corresponding to false iodine signals nearly half as strong as a true 2‐mg/mL enhancement. Residual error was computed using the mean signal across all noniodine ROIs; lung‐only estimates would be higher due to stronger false positives in low‐density regions. In clinical terms, this may mask hypoperfused myocardium adjacent to calcified coronaries, create pseudo‐enhancement in calcified lung nodules, or generate false‐positive pulmonary emboli from calcified hilar nodes. PCA‐MMD's reduction to 1.6% residual effectively eliminates these diagnostic pitfalls, enabling confident interpretation even in heavily calcified thoraces. This reduction was highly significant (p<0.001). In addition to false‐positive residuals, we examined potential false‐negative iodine estimates, defined as the suppression of a true iodine signal in mixed water–lipid–iodine voxels due to partial‐volume effects. Because DECT provides limited information, such bias can arise when the true mixture is not explicitly represented in the selected basis‐material library and domain partitioning. Importantly, this effect depends primarily on the library configuration and domain selection strategy rather than the solver itself. Since physical phantoms cannot realize controlled sub‐voxel mixtures, we performed a numerical sensitivity analysis using synthetic three‐material mixtures generated by linearly combining the attenuation coefficients of water, lipid, and iodine. Under the default five‐domain library, a representative 40% water, 40% lipid, 20% iodine mixture was assigned to the (water–air–iodine) domain, yielding an iodine estimate of 0.15 versus the true 0.20 (25% underestimation). To illustrate the effect of library configuration, we evaluated an augmented library including an additional (water–lipid–iodine) domain using a synthetic example. Under this augmented library, the same mixture was correctly assigned by the existing centroid‐based domain selection strategy (Pass 1, Section 2.5.2), and the iodine estimate matched the ground truth. Under this augmented library, the same voxel was correctly assigned and the iodine estimate matched the ground truth, demonstrating that PCA‐MMD can mitigate false‐negative iodine suppression through application‐specific library extension without compromising numerical stability or physical plausibility.

Size‐dependent analysis showed degraded accuracy for both methods with increasing phantom size, consistent with increased scatter and beam hardening. However, PCA‐MMD exhibited a more gradual degradation than BC‐MMD (Figures 4 and 6), suggesting improved robustness to size‐related confounding effects through the PCA transformation.

Most prior iodine‐quantification studies have focused on EID‐based DECT systems and intervendor comparisons. Jacobsen et al.^66^ reported iodine errors of 0.44–1.70 mg/mL across seven DECT scanners, with reduced accuracy at low concentrations where quantum noise dominates. Multiple studies further reported size‐ and dose‐dependent degradation and visible residual artifacts in larger phantoms.^18^, ^67^, ^68^, ^69^ More recently, Winfree et al.^26^ and Salyapongse et al.^70^ showed that PCCT generally improves iodine quantification, although EID systems remain the clinical workhorse. To address generalizability, we performed a proof‐of‐concept validation on a mainstream EID system. As detailed in the Supplementary Material (Table S1), PCA‐MMD demonstrates feasibility on the EID platform, achieving statistically significant reductions in RMSE and residual errors relative to the baseline method (p<0.05). These findings suggest that the performance gains are attributable to the geometric PCA‐based framework rather than being solely dependent on the superior spectral purity of PCCT. To the best of our knowledge, the present study is the first to comprehensively evaluate iodine quantification accuracy and residual errors across multiple dose levels and phantom sizes using a clinical PCCT system. Furthermore, we introduce a novel PCA‐MMD method that is both accurate and computationally efficient (summarized in Table 3), achieving full‐volume decomposition of 11.8M voxels in 0.44 s using GPU acceleration—making it well suited for clinical implementation.

While phantom studies provide a controlled environment for quantitative benchmarking, they lack the anatomical complexity, biological variability, spectral distortions encountered in vivo, and physiological motion inherent in clinical imaging.^71^, ^72^ Therefore, we evaluated PCA‐MMD in clinical patient studies and observed improved performance relative to both BC‐MMD and a proprietary vendor‐specific algorithm implemented in the commercial Syngo.via platform (Siemens Healthineers). PCA‐MMD yielded VNC images that were statistically closer to the TNC reference, achieving a mean RMSE of 15.5 HU compared to 20.9 HU for the vendor algorithm (p=0.012) and 25.4 HU for BC‐MMD (p<0.001) (Table 4). This validation strategy aligns with recent studies by Risch et al.^73^ and Steinhardtet al.,^74^ which have established paired TNC–VNC comparisons as the standard metric for assessing spectral reconstruction accuracy in the absence of histological ground truth. The improved in vivo performance suggests robustness to spectral skewing from scatter and beam hardening.^75^ By adaptively identifying the iodine vector via PCA, the proposed framework better accommodates local background variability than the fixed basis constraints of BC‐MMD, resulting in a 56% reduction in residual error. It is also worth noting that we observed differences in image texture within the cardiac chambers between the vendor VNC and the PCA‐MMD and BC‐MMD methods (Figure 8). This is because the evaluated PCA‐MMD and BC‐MMD approaches perform independent voxel‐wise decompositions, preserving the underlying quantum noise texture, whereas commercial implementations often incorporate proprietary spatial regularization or noise‐reduction strategies to enhance visual smoothness.

### 4.1 Limitations

This study has a number of limitations. First, the proposed PCA‐MMD framework was benchmarked primarily against a single well‐known MMD approach.^20^ While this comparison provides a relevant baseline for evaluating improvements in residual suppression and quantification accuracy, comparisons with additional spectral CT decomposition techniques would provide a broader evaluation of algorithm performance. Second, while our framework is designed to be robust by partitioning the attenuation space into multiple, physically motivated domains, quantification accuracy within each domain remains dependent on the initial choice of basis materials. As reported by Salyapongse and Szczykutowicz,^75^ even mathematically correct decompositions can be influenced by the selected basis if it does not perfectly represent the true object composition. Thus, although our framework mitigates reliance on a single ill‐fitting basis, future work should assess sensitivity to variations in the material library (e.g., substituting collagen with alternative protein surrogates) and explore strategies to optimize basis selection. Third, in the clinical proof‐of‐concept, absolute ground truth material maps (e.g., histology or chemical assay) were unavailable. We used TNC images as the reference for VNC accuracy; while widely accepted, this evaluates the combined noniodine components rather than individual material maps. Fourth, while the clinical sample size of five subjects was sufficient for a proof‐of‐concept evaluation and to demonstrate statistically significant improvements, consistent with prior proof‐of‐concept studies,^25^ the cohort may not be sufficiently large or heterogeneous to fully represent broader patient populations. Consequently, the generalizability of the clinical findings remains limited. Future work will therefore require larger‐scale validation across more diverse patient populations, body habitus, and pathological conditions.

## 5 CONCLUSION

This study addressed key limitations in DECT MMD, including inaccurate iodine quantification and residual errors that reduce diagnostic confidence, particularly under low‐dose conditions. We introduced and validated the PCA‐MMD method, demonstrating promising performance across multiple phantom sizes and clinically relevant dose levels. The primary contribution of PCA‐MMD is integrating PCA with a geometrically constrained, multipass decomposition strategy. By transforming attenuation data into a PCA‐aligned space, the framework improves material separability and numerical stability, enforces physically plausible volume fractions by construction, and mitigates the instability and bias common in conventional approaches. Phantom experiments showed reduced residual iodine contamination and improved quantification accuracy, while GPU acceleration enabled rapid full‐volume decomposition suitable for clinical workflows. Clinically, improved robustness at reduced dose levels supports broader DECT use in dose‐sensitive applications, and a proof‐of‐concept patient evaluation demonstrated improved VNC accuracy compared with both the baseline method and a commercial vendor algorithm. Overall, PCA‐MMD provides a physically grounded, stable, and computationally efficient approach for DECT MMD, motivating future large‐scale clinical validation.

## CONFLICT OF INTEREST STATEMENT

The authors declare no conflicts of interest.

## Supporting information

Supporting Information
