# Supplementary material for: Accurate iodine quantification and residual error reduction with principal component analysis multimaterial decomposition using spectral CT
Source: Med Phys. 2026 Apr 10;53(4):e70407. doi: 10.1002/mp.70407 (PMC13067357; doi:10.1002/mp.70407)
Supplement: Supplementary file 1 — Supporting Information [file MP-53-0-s001.pdf]

# Supplementary Material

## S1 Proof-of-Concept Evaluation on an Energy-Integrating Detector System

### S1.1 Methodology

To evaluate the generalizability of the proposed PCA-MMD algorithm beyond PCCT systems, a proof-of-concept study was conducted on a mainstream energy-integrating detector (EID) dual-energy CT system. The same anthropomorphic thorax phantom as described in Section 2.1, configured with the average adult build (water-equivalent diameter, $D_w \approx 28$ cm, defined as the reference patient size [76]), was scanned on a 256-slice MDCT scanner (Revolution CT, GE Healthcare, Waukesha, WI, USA) equipped with Gemstone Spectral Imaging (GSI) technology for fast kVp-switching dual-energy acquisition. This phantom size was selected to approximate the average water-equivalent diameter observed in adult patients undergoing cardiac or chest CT examinations, where reported values typically range from 20 to 30 cm in clinical cohorts [77, 78]. The scan employed a routine cardiac GSI protocol with fast kVp switching between 80 and 140 kVp, yielding a site-representative $\text{CTDI}_{\text{vol}}$ of approximately 12 mGy. This dose level aligns with reported clinical protocols for dual-energy coronary CT angiography on GE fast kVp-switching systems, where $\text{CTDI}_{\text{vol}}$ values for standard-sized patients commonly range between approximately 10 and 20 mGy [79, 80, 81]. VMIs were reconstructed on the scanner at 70 keV and 140 keV from the GSI dataset to provide low- and high-energy inputs for both the BC-MMD and the proposed PCA-MMD algorithms. Adaptive statistical iterative reconstruction (ASiR-V at 50%) was applied for image reconstruction. The slice thickness was maintained at 3 mm, consistent with our selection in the main PCCT study. Quantitative analysis of iodine concentration accuracy and residual error maps followed the same methodology outlined in Section 2.6, including region-of-interest measurements and statistical comparisons.

### S1.2 Results

This proof-of-concept experiment was designed to test whether PCA-MMD performance gains are dependent on PCCT-specific spectral properties, using a representative adult-sized phantom and a routine clinical EID acquisition protocol (Section S1.1).

**Table S1:** Proof-of-concept quantitative comparison of BC-MMD and PCA-MMD on an EID-based dual-energy CT system (GE Revolution CT).

| **Metric** | **BC-MMD** | **PCA-MMD** | **Error reduction (%)** |
|---|---|---|---|
| RMSE (mg/mL) | 0.58 | 0.28 | 52% |
| Residual error (%) | 30.5 | 3.2 | 90% |

* Statistically significant differences in quantification accuracy and residual error were observed ($p < 0.05$).

The quantitative comparison, summarized in Table S1, demonstrates improved performance of PCA-MMD relative to the conventional BC-MMD method. Specifically, PCA-MMD achieved a lower iodine quantification error, with an RMSE of 0.28 mg/mL compared to 0.58 mg/mL for BC-MMD (52% reduction). In addition, PCA-MMD substantially reduced residual iodine contamination in non-iodine regions, decreasing

the residual error from 30.5% to 3.2% (90% reduction). Statistical analysis confirmed that improvements in both accuracy and residual suppression were significant ($p < 0.05$).

These results indicate that the proposed PCA-MMD framework can be deployed on a mainstream EID-based dual-energy CT system without requiring scanner-specific parameter tuning, while consistently outperforming the baseline BC-MMD approach under clinically relevant conditions.
